# *Chlamydomonas reinhardtii,* a Reference Organism to Study Algal–Microbial Interactions: Why Can’t They Be Friends?

**DOI:** 10.3390/plants12040788

**Published:** 2023-02-09

**Authors:** Victoria Calatrava, Manuel Tejada-Jimenez, Emanuel Sanz-Luque, Emilio Fernandez, Aurora Galvan, Angel Llamas

**Affiliations:** 1 Department of Biochemistry and Molecular Biology, Campus de Rabanales and Campus Internacional de Excelencia Agroalimentario (CeiA3), Edificio Severo Ochoa, University of Córdoba, 14071 Córdoba, Spain; 2 Department of Plant Biology, Carnegie Institution for Science, 260 Panama St., Stanford, CA 94305, USA

**Keywords:** microalga, *Chlamydomonas*, biotic interactions, algal–microbial consortia

## Abstract

The stability and harmony of ecological niches rely on intricate interactions between their members. During evolution, organisms have developed the ability to thrive in different environments, taking advantage of each other. Among these organisms, microalgae are a highly diverse and widely distributed group of major primary producers whose interactions with other organisms play essential roles in their habitats. Understanding the basis of these interactions is crucial to control and exploit these communities for ecological and biotechnological applications. The green microalga *Chlamydomonas reinhardtii*, a well-established model, is emerging as a model organism for studying a wide variety of microbial interactions with ecological and economic significance. In this review, we unite and discuss current knowledge that points to *C. reinhardtii* as a model organism for studying microbial interactions.

## 1. Why Microalgae and Why *Chlamydomonas reinhardtii*?

Microalgae are a highly diverse group of unicellular and photosynthetic eukaryotes that include major primary producers on our planet. Although highly abundant in aquatic ecosystems, microalgae can live in many habitats, including on the poles, below the ground within the crowded rhizosphere, or inside animal cells in coral reefs. These microbes can have photoautotrophic, heterotrophic, or mixotrophic lifestyles, show a broad range of cell sizes, morphologies, architectures, and exhibit a highly diverse metabolic capacity that offers several unique features for scientific research [1]. Responsible for a significant amount of total global carbon (C) fixation [2], microalgae are crucial to sustaining the ecosystems, but can also cause their disruption during algal blooms, which can pose a great ecological, economical, and health threat [3]. Furthermore, the use of microalgae in biotechnological applications has exploded in this century. For example, they are used in wastewater treatment, biofuel production, animal feed, or high-value compounds, and are promising organisms to increase biological C sequestration to mitigate global warming [4]. Therefore, microalgae are of tremendous ecological and economic interest.

Although polyphyletic, microalgal members are related by the ancestral origin of their plastid, which was initially the result of a primary endosymbiotic event involving a cyanobacterium that evolved into the plastid of the common ancestor of the Archaeplastida (i.e., glaucophytes, red and green algae, and land plants); and subsequently, expanded from red and green algae to unrelated eukaryotes through secondary and tertiary endosymbiosis, giving rise to the exceedingly colorful and metabolically diverse extant algal groups (e.g., diatoms and dinoflagellates) [5]. 

Over the centuries, microalgae have been isolated from nature—many of which have failed to grow axenically. Their reliance on other microorganisms is likely a consequence of the long-term co-evolution of microalgae with their symbiotic microbes [6]. Microalgae can form symbiotic relationships with a wide variety of organisms, including bacteria, fungi, plants, and animals [7]. When mutualistic, microalgae generally provide their partner(s) with fixed C and oxygen in exchange for limiting nutrients and essential molecules, such as available forms of vitamins and nitrogen (N) [8,9]. Additionally, a much broader set of molecules can be secreted, perceived, and utilized by the interacting partners. The elucidation of the intricacy and dynamics of many of these symbioses has only just begun [10,11]. 

The microalga *C. reinhardtii* (hereafter *Chlamydomonas*) was first isolated in 1945 from the soil of a potato field in Massachusetts (U.S.A.) and has since become a powerful model organism [12]. This green alga, sharing a common ancestor with land plants, has fundamental features related not only to plants but also to animals [13]. Some of the research topics that are being addressed using *Chlamydomonas* are photosynthesis, respiration, the metabolism of nitrogen (N), sulfur, phosphorus, amino acids and metals, the biosynthetic pathways of starch, lipids, carotenoids, hemes groups, glycerolipids, chlorophyll, and other fundamental aspects including the function of chaperones, proteases, thioredoxins, cilia biogenesis, and the responses to different stress conditions [14]. 

There are many features that make *Chlamydomonas* an excellent microbial model, as recently reviewed by Salomé *et al.* [15]. It grows well axenically, has a relatively rapid doubling time (~8–12 h), and the sequences of its nuclear, chloroplast, and mitochondrial genomes are available. In addition, *The Chlamydomonas Sourcebook* [12] provides a compendium of the major research lines, history, physiology and methodology of *Chlamydomonas*, and ‘The *Chlamydomonas* Resource Centre’ (www.chlamycollection.org, accessed on 7 February 2023) has numerous tools available, including biochemical assays, protocols, plasmids, and thousands of mapped mutant strains. Furthermore, the development of various techniques for gene editing in *Chlamydomonas*, such as CRISPR-Cas9, represents a decisive advance for addressing both fundamental questions and biotechnological applications [16,17].

Since its discovery, the number of reports studying *Chlamydomonas* interactions with diverse microorganisms has exponentially increased. Here, we summarize and categorize those reports into *Chlamydomonas* predators, fungal partners, and bacterial mutualists, and discuss the potential of microbial interactions for biotechnological applications. 

## 2. Biodiversity in the *Chlamydomonas* Phycosphere

**Figure 1:**
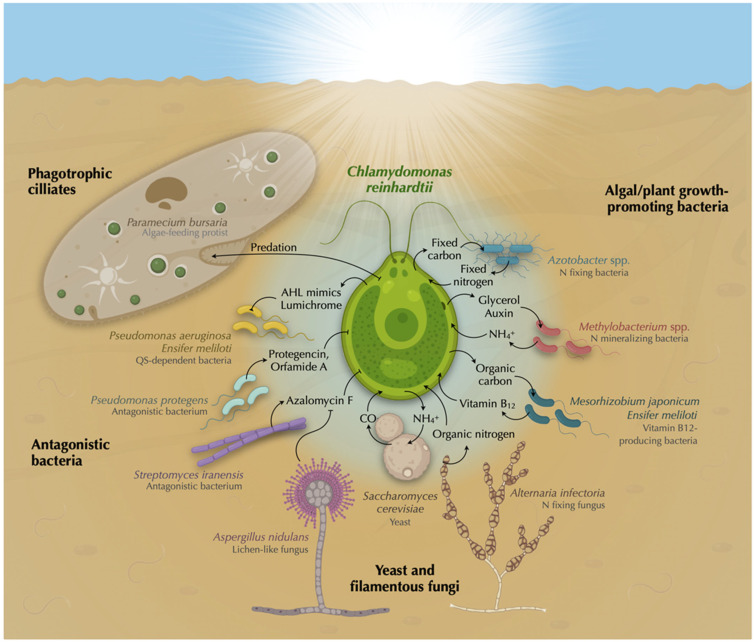
**Interactions between *Chlamydomonas reinhardtii* and other microbes, illustrating the main topics covered in this review**. This illustration represents a *Chlamydomonas* cell in the central part surrounded by its phycosphere populated by representative interacting microbes and indicating the main molecules involved in the interaction. *Chlamydomonas* is prey for a variety of different microbes, including protists such as *Paramecium bursaria* [37], *Tetrahymena vorax* [32], and *Peranema* [14], and soil bacteria such as *Streptomyces iranensis* [33] and *Pseudomonas protegens* [34,35,36]. The bacterium *P. protegens* produces an arsenal of anti-algal compounds and secondary metabolites such as orfamide A and protegencin that induce morphological changes, inhibit growth, and can kill *Chlamydomonas* [35,36]. *S. iranensis* produce the algicidal compound Orfamide A in the presence of *Chlamydomonas,* which can be physically protected from the algicide by the lichen-like fungi *Aspergillus nidulans* [33]. *Chlamydomonas* produces at least a dozen AHL mimics that can stimulate QS in the soil-dwelling bacterium *Ensifer meliloti* [106]; among them, lumichrome was able to stimulate the QS LasR receptor in *Pseudomonas aeruginosa* [109]. *Chlamydomonas* establishes spontaneous obligate mutualisms with fungal species under specific conditions [46]. In closed systems under illumination with glucose and nitrite, *Saccharomyces cerevisiae* metabolizes glucose to feed with CO_2_ *Chlamydomonas*, which provides the yeast with ammonium produced from nitrite. The ascomycetous fungus *Alternaria infectoria* fixes N_2_ that can be supplied to *Chlamydomonas* in the form of organic nitrogen, and they can form long-living consortia [47]. The rhizospheric bacteria *Mesorhizobium japonicum* and *Ensifer meliloti* can provide *Chlamydomonas* with vitamin B_12_ promoting algal growth in exchange for algal fixed C [50,58]. Nitrogen mineralizing bacteria such as *Methylobacterium* spp. can break down amino acids and peptides to feed *Chlamydomonas* with ammonium in exchange for the photosynthate glycerol [87]. Additionally, auxin produced by *Chlamydomonas* can feed *Methylobacterium aquaticum*, which in turn promotes algal growth [99]. Lastly, N_2_-fixing bacteria such as *Azotobacter chroococcum*, *A. beljerinckii*, *A. agilis,* and *A. vinelandii* can support *Chlamydomonas* growth with fixed nitrogen in exchange for fixed carbon [65,67,68,69,71]. This figure was generated using Biorender.com and Keynote software.

The phycosphere (Figure 1), the algal analog of the plant rhizosphere, has been defined as the region immediately surrounding algal cells, covering an undefined distance between the algal outer membrane and the aqueous zone in which bacterial growth is stimulated by extracellular algal products [18,19]. 

Microalgae produce large amounts of dissolved organic matter (DOM) that is released and accumulated in this microscopic environment, feeding the microbes inhabiting this niche. The bacterial communities in the phycosphere are taxonomically different to those in algal-free environments, similar to the differences observed between rhizosphere and bulk soil bacteria [20]. The delivery of DOM via photosynthate seems to be important for microalgae–bacterial interactions, and some other factors provided by the bacteria may facilitate and enhance their relationships [21]. Some studies have focused on characterizing the phycosphere microbiota to identify and understand the relevant interactions between microalgae and bacteria in their natural environment [22,23]. Thus, the phycosphere of different microalgae with great potential in the industry, including *Chlamydomonas* and other green algae such as *Chlorella vulgaris, Scenedesmus* sp., and *Botryococcus braunii,* has been characterized [24]. In this study, the phycosphere of *Chlamydomonas* was found to be dominated by *Hyphomicrobiaceae sp*. (44%), *Mesorhizobium sp.* (15%), and *Rhizobium sp.* (12%) [24]. 

Although bacterial associations can be species-specific, the analysis of the phycosphere of several phylogenetically distant green algae isolated from different environments revealed that most closely associated bacteria belonged to the phyla Bacteroidetes and α-Proteobacteria, which include members known to be symbiotic with plants. These observations suggested that at least some of these bacteria may have co-evolved with their green photosymbionts [6]. Supporting this idea, a more recent study comparing *Chlamydomonas* phycosphere microbiota with the root microbiota of *Arabidopsis thaliana* has shown an extensive taxonomic overlap, including *Rhizobia*, *Pseudomonas*, *Burkholderia*, and *Xanthomonas* spp. [23]. Moreover, the most abundant bacterial orders found in this comparison were shared between the root communities of different land plants and the phycosphere of several green algae, further supporting their possible co-evolution. This indicates that despite the vast evolutionary distance between *Chlamydomonas* (chlorophyte) and *A. thaliana* (embryophyte), the assembly of their microbiota might be driven by shared ecological principles that are not yet fully understood. Nevertheless, host specificity was also observed for these associated bacteria, as they can outcompete non-native strains only in the presence of their corresponding host. This study shows that establishing the phycosphere microbiota requires physical proximity between *Chlamydomonas* and its microbiota, probably because a bi-directional exchange of metabolites is required for their assembly [23]. 

*Chlamydomonas* has proven to sustain C cycling with a variety of taxonomically distinct bacteria isolated from soil [25]. Using Closed Microbial Ecosystems (CES) in which microbial communities are hermetically closed and maintained with only light, *Chlamydomonas* can feed the bacterial community with fixed C. In turn, the community produces CO_2_ to feed the alga, resulting in the self-organization of the consortia and robust C cycling to support the closed community for up to 50 days. In contrast, *Chlamydomonas* failed to persistently cycle C with *Escherichia coli* in the same closed system, likely due to *E. coli’s* inability to assimilate the starch produced by *Chlamydomonas* [25]. However, other studies using CES with the ciliate predator *Tetrahymena thermophila* in co-culture with *Chlamydomonas* and *E. coli* have shown that these three species can be maintained for years [26,27]. This suggests that introducing a phagotroph that can feed on the bacteria and, occasionally, on the algal population, allows for successful C cycling in this closed ecosystem. Additionally, *Chlamydomonas* can prevent bacterial invasion of *T. thermophila* cultures by inhibiting aggregation in *E. coli*—a defense mechanism to avoid predation—thus controlling the bacterial population by increasing predation pressure on *E. coli,* which is caused by the ciliate [28].

Even though the studies focused on characterizing *Chlamydomonas’* microbiota are scarce and mostly limited to bacteria, this microalga shares a habitat with many other microbes, including other protists, fungi and viruses, that are likely relevant for *Chlamydomonas’* biology in nature.

## 3. Chlamydomonas’ Predators

In addition, as a primary producer, *Chlamydomonas* not only feeds other organisms by secreting fixed C, but also by serving as food when lysed or ingested by predators. *Chlamydomonas* is a form of prey for a variety of different organisms, including rotifers such as *Brachionus calyciflorus* [29], crustaceans as *Daphnia* [30,31], protists such as *Tetrahymena vorax* [32] and *Peranema* [14], and soil bacteria such as *Streptomyces iranensis* [33] and *Pseudomonas protegens* [34,35,36] (Figure 1). 

The bacterium *P. protegens* produces an arsenal of anti-algal compounds—secondary metabolites that induce morphological changes, inhibit growth, and can kill *Chlamydomonas* [35]. For instance, orfamide A induces an increase in cytosolic calcium levels in *Chlamydomonas* that causes deflagellation, whereas protegencin causes destruction of the carotenoids and eventually lyses algal cells [36] (Figure 1). This toxicity by protegencin also affects an eyeless mutant of *Chlamydomonas* and its colony-forming relative, *Gonium pectorale*. The protegencin biosynthesis gene cluster is ubiquitous in *Pseudomonas* genomes, which suggests that this antagonistic interaction is likely ecologically relevant between green algae and *Pseudomonas* species [36]. Interestingly, a *P. protegens* mutant impaired in secondary metabolism not only loses its anti-algal activity, but may promote *Chlamydomonas* growth [35].

The phagotrophic ciliate *Paramecium bursaria*, bearing the endosymbiotic green alga *Chlorella*, can feed on *Chlamydomonas* cells, which benefit both the host and endosymbiont by providing nutrients and clearing competitors for the latter [37] (Figure 1). To avoid being ingested by predators, *Chlamydomonas* can form cell aggregates of 4–16 cells, named palmelloids, and larger aggregates of up to thousands of cells [38]. The formation of palmelloids were the result of successive divisions induced by stress without degradation of the cell wall [39] and can be induced by predators such as the rotifer *Brachionus calyciflorus* [29] and the ciliate *Paramecium tetraurelia* [40,41]. Importantly, continuous exposure to predators repeatedly induced heritable genetic changes in *Chlamydomonas*, supporting the hypothesis that predation may have been one of the driving forces for the evolution of multicellularity [40,41,42].

The phagotrophic euglenoid *Peranema trichophorum* can also induce different *Chlamydomonas* spp. to form cell aggregates of up to 10,000 cells, composed of the same or different species, that dissociated when the predator was removed [43]. Interestingly, the culture filtrate of *P. trichophorum* also induces algal cell aggregation, likely in response to putative chemicals secreted by the predator that are still unknown (i.e., kairomones) [43]. Other kairomones produced by *Daphnia* and *Daphnia*-feeding fish can also alter *Chlamydomonas* behavior by generally inducing phototaxis (i.e., motility in response to light) [44]. The detection mechanism of kairomones in *Chlamydomonas* and whether it is predator-specific remains unclear.

## 4. *Chlamydomonas’* Fungal Partners

Lichens, one of the most studied examples of obligate mutualisms, are formed by a self-sustaining association of fungi (mostly ascomycetes) with green algae, although they can also occur with cyanobacteria, as well as with bacteria and yeasts [45]. The study of synthetic consortia has revealed that *Chlamydomonas* can establish spontaneous obligate mutualisms with different fungal species—including the yeast *Saccharomyces cerevisiae,* and the filamentous fungi *Neurospora crassa* and *Aspergillus nidulans*—under specific conditions [46]. In closed systems, under illumination and in the presence of glucose as the sole C source, which cannot be used by *Chlamydomonas,* and nitrite as the only N source, which cannot be assimilated by the yeast *S. cerevisiae*, the alga and the yeast metabolize and cross-feed available sources of N and C; *S. cerevisiae* metabolizes the glucose, releasing carbon dioxide that is used by *Chlamydomonas*, which in turn reduces nitrite to feed the yeast with ammonium. This synthetic consortium has also proven relevant to assess the role of ecological interactions in the evolution of mutualism; the presence of the alga shifts natural selection to fix mutations in the yeast that strengthen their mutualistic interaction [10].

*Chlamydomonas* cells were found to physically interact with the filamentous fungi *Neurospora crassa* and *Aspergillus nidulans*, to likely facilitate nutrient exchange [46]. Moreover, *Chlamydomonas* and the ascomycetous fungus *Alternaria infectoria* can form long-living consortia that persist for up to several years in the absence of a N source other than atmospheric N_2_, which was fixed by the fungus and likely supplied to the alga as amino acids [47]. 

Additional beneficial interactions with the lichen forming fungus *A. nidulans*, which chemoattracts *Chlamydomonas*, include physical protection against the algicidal compound Azalomycin F produced by the bacterium *Streptomyces iranensis* in the presence of *Chlamydomonas* [33]. The shelter provided by the fungus suggests that the formation of algal–fungal associations may be a good strategy against harmful microorganisms and may have driven the evolution of lichen associations [33].

These systems have provided important clues about the possible abiotic and biotic conditions that initially led to the evolution of lichens and revealed valuable insights into the emergence of symbiosis and the design of synthetic biological systems.

## 5. *Chlamydomonas’* Bacterial Partners

### 5.1. Vitamin B_12_ Production

Vitamin B_12_ (cobalamin) is an enzyme cofactor synthesized only by prokaryotes, yet more than half of microalgae require it for growth [8]. Therefore, this cofactor may act as a common bacterial currency for mutualism with algae in exchange for algal fixed C, and it may play a key role in microbial community structure and function [48]. Nevertheless, not all B_12_-producing bacteria and algal B_12_ auxotrophs establish mutualistic relationships, indicating a species-specificity that may be due to the amount of B_12_ released or the type of C fixed secreted by the alga [49,50]. While some studies have shown that bacterial growth was required to support algal B_12_ auxotrophs [51], others have reported that bacterial cell lysis was sufficient for that purpose [49]. In algae, the enzyme methionine synthase (*METH*) uses vitamin B_12_ as a cofactor, but some algae, such as *Chlamydomonas*, also have a B_12_-independent isoform (*METE*) that allows them to grow without the vitamin, albeit less efficiently [52]. Under heat stress, the *Chlamydomonas METE* gene is repressed, leading to chlorosis and cell death if this vitamin is unavailable. However, addition of exogenous B_12_ or co-culture with the B_12_-producing bacterium *Ensifer meliloti* allows *Chlamydomonas* to thrive under heat stress [53], indicating that abiotic stress could promote such beneficial interactions in nature [53] (Figure 1).

The loss of the *METE* gene, which leads to B_12_ auxotrophy, appears to be independent in the evolutionary history of algae, as it seems to have occurred several times [54,55]. Indeed, a B_12_ auxotroph of *Chlamydomonas* was generated via experimental evolution after ca. 500 generations in B_12_-replete media [52]. This newly evolved B_12_-dependent strain was then able to grow in the absence of this vitamin when co-cultured with B_12_-producing bacteria, including the rhizobium *Mesorhizobium japonicum* or even an *E. coli* strain engineered to produce and release B_12_ [50]. Nevertheless, it is unclear whether the B_12_-dependent strain would survive in nature. On the one hand, this strain grew more slowly in laboratory co-culture with bacteria than the B_12_-independent parental strain, but on the other hand, experimental co-evolution with bacteria improved the alga’s subsequent growth in co-culture and may suggest that prolonged coexistence with bacteria in the environment could favor the evolution of B_12_ auxotrophy [50]. 

Supporting this idea, Kazamia and collaborators found that *M. japonicum* established a mutualistic relationship based on B_12_-C exchange with the naturally B_12_-dependent green alga *Lobomonas rostrata*, which is closely related to *Chlamydomonas* [56]. Furthermore, proteomic analyses of this mutualism suggested that amino acids may be the reduced form of C that the algae were feeding the bacterium in exchange for B_12_ [57].

More recently, the integration of time-resolved isotope labeling followed by secondary ion mass spectrometry (SIMS) method with mechanistic modeling, has elucidated the role of nutrient exchange in controlling the inception and temporal onset of a mutualistic relationship between the B_12_ auxotrophic *Chlamydomonas* mutant *mete7* and *M. japonicum* [58]. The results showed that these microbes, which were not given the time to co-evolve and whose growth does not seem to be induced by the interaction, established a mutualism based on the exchange of vitamin B_12_ and organic C. The combination of nutrient measurements and mathematical models might be a useful tool for the mechanistic understanding of more complex microbial interactions, such as those composed of more than two partners [58,59].

### 5.2. Nitrogen Fixation

N is highly abundant in the atmosphere as dinitrogen gas (N_2_), but it is only accessible to some prokaryotes (diazotrophs) which are capable of converting it to the fixed N form ammonium. Therefore, N-limited photoautotrophic eukaryotes often partner with diazotrophs that provide them with usable N sources, forming stable symbioses. One of the most extensively studied symbioses is that established between land plants (legumes) and symbiotic diazotrophs (rhizobia), which provide the plant with fixed N in exchange for nutrients and energy [60]. Although far less studied, diazotrophs can also form a symbiosis with different algae, including diatoms [61,62], haptophytes [63], and green algae [64,65].

The free-living symbiotic diazotroph *Azotobacter chroococcum* has been widely used as biofertilizer, successfully increasing the yields of a wide variety of crop plants [66]. Moreover, *Azotobacter* species can also support the growth of different algal species, including *Chlamydomonas*. In media without any N or C sources, spontaneous mutualisms have been observed by mixing different *Azotobacter* species (*A. chroococcum*, *A. beljerinckii*, *A. agilis*, *A. vinelandii*) with *Chlamydomonas* [67,68] (Figure 1). These algal–diazotroph co-cultures survived for several years, and algal photosynthetic efficiencies were observed to be similar to those growing with N, which strongly suggests that the diazotroph provides the alga with readily available N source(s) [69]. Additionally, these bacteria can provide the alga with amino acids, vitamins, and hormones, which can deliver proteins, polysaccharides, and glycolate to the bacterium in exchange [70]. While this association was extracellular, cell-to-cell fusion was artificially induced with polyethylene glycol to force the endosymbiotic interaction of *Azotobacter* cells within *Chlamydomonas* that were able to grow in N- and C-free medium for several years [71]. In this alga, *Azotobacter* cells have been found in organelle-resembling vesicles located in the cytoplasm, between the cell wall and plasma membrane, across the cell wall, and in the periplasmic space. The number of symbionts in these cellular locations was found to be regulated, and the *Chlamydomonas*–*Azotobacter* volume ratios were nearly constant (47.2 ± 2.4), which may be key to the stabilization of the interaction [72]. To achieve the establishment of the co-culture, the depletion of C and N in the media seems to be critical, although the mechanism that controls the algal–bacterial volume ratio and the specific C and N compounds exchanged between these organisms remains elusive [65]. Although it seems clear that the C-fixing alga must be providing the heterotrophic bacterium with fixed C, and the N-fixing bacterium must be feeding the alga with fixed N, the exact C and N compounds that are exchanged are unknown. *A. vinelandii* excretes 17 amino acids [65], many of which can support *Chlamydomonas* growth after their deamination [73], while *Chlamydomonas* can secrete different C compounds that could serve as C and energy sources for the bacterium. Some of these compounds were glycolate; oxalate; pyruvate [74]; acetate, which was more efficiently used by *Azotobacter* than glucose [75]; several keto acids [76]; amino acids and sugars [77]; and various fermentation products such as acetic acid, formic acid, ethanol, and malic acid [78].

Resembling the plant symbioses with mycorrhizal fungi and diazotrophic bacteria, which improve their growth through the mobilization of nutrients [79], an artificial tripartite symbiosis involving *Chlamydomonas, Azotobacter,* and the fungus *Alternaria* has been found to support algal growth for three years on a N and C-free medium [80]. In this system, the fungus can secrete a wide range of amino acids, increasing the diversity of N compounds available for the alga, compared to those secreted by bacteria in the dual system, and positively impacting algal survival [68]. The sulfur-containing amino acid and metabolic intermediate cystathionine was found to be highly secreted by the fungus and increased in the tripartite system [80], likely providing sulfur to the alga and/or bacterium. Curiously, a cystathionine-β-synthase-like protein was required for rhizobial infection and symbiotic N fixation in *Medicago truncatula* [81]. However, whether the production of cystathionine by *Alternaria* is involved in the establishment of the tripartite association with *Chlamydomonas* and *Azotobacter* awaits further examination.

### 5.3. Amino Acids and Peptides Mineralization

In environments or conditions in which inorganic N is limiting but organic N is abundant, plant growth-promoting bacteria (PGPB) play a critical role in mineralizing organic N to inorganic forms, which are assimilable by photosynthetic organisms [82]. As previously mentioned, amino acids can act as a microbial currency to establish symbiotic interactions with *Chlamydomonas.* Although less efficiently than inorganic N forms such as ammonium or nitrate, this alga can metabolize a wide variety of amino acids and peptides as the sole N source [83]. This growth mainly relies on the extracellular activity of an L-amino acid oxidase encoded by the *LAO1* gene and a specific high-affinity uptake system for L-arginine [73,84,85]. However, this alga cannot utilize the amino acid L-proline as well as short peptides as N sources [86]. The PGPB *Methylobacterium* species can “rescue” *Chlamydomonas* growth on L-proline as the only N source, establishing a mutualism. This mutualistic interaction is species-specific and based on a C-N exchange: the bacterium metabolizes L-proline, producing ammonium to feed *Chlamydomonas*, which releases the photosynthate glycerol to feed the bacterium [87] (Figure 1), although additional metabolites may also be exchanged. In addition to L-proline, *Methylobacterium* spp. were able to support algal growth on a variety of di- and tri-peptides containing L-alanine, glycine, L-proline and L-hydroxyproline [87]. Interestingly, the cell wall of *Chlamydomonas* is enriched in glycoproteins containing proline and hydroxyproline [88] and may secrete L-proline [80], which might be used to attract beneficial *Methylobacterium* spp. [87].

Although *Chlamydomonas* can grow on most free amino acids through the LAO1 enzyme, this extracellular activity may represent a beneficial strategy for microbial interactions [73]. LAO1 breaks down amino acids into the corresponding keto acid, H_2_O_2_ and ammonium [84], which is very efficiently taken up by the alga through high-affinity transport systems [89]. The remaining byproducts, keto acids and H_2_O_2_, were accumulated in the phycosphere and might serve as public goods for other microorganisms [73]. In the phycosphere, keto acids can be used as a C and an energy source by other microorganisms, play a role in solubilizing or chelating iron, buffer the toxic H_2_O_2_ [90], and help algal cells to establish interactions with other organisms. In fact, the keto acid indole-3-pyruvic acid, generated from L-tryptophan, was found to be a major intermediate of the auxin indole-3-acetic acid, and its impact on mutualism is discussed below (Section 5.4). The highly reactive H_2_O_2_ produced by LAO1 could aid *Chlamydomonas* to avoid competitors and promote the growth of beneficial bacteria that specifically contain H_2_O_2_-detoxifying enzymes such as catalase (e.g., *Methylobacterium* spp.). Supporting this idea, H_2_O_2_-consuming bacteria were present in the natural habitat of the diatom *Amphiprora kufferathii*, which can eliminate the H_2_O_2_ produced by the algal cells [91]. Similarly, other prokaryotes, such as *Prochlorococcus* spp., *Sinorhizobium meliloti*, and *Vibrio fischeri*, have shown mutualistic interactions based on H_2_O_2_ detoxification [92,93,94], suggesting that this is a widespread mechanism of symbiotic relationships.

### 5.4. Auxin Production and Degradation

The plant auxin indole-3-acetic acid (IAA) is a major signaling molecule involved in controlling plant–bacterial interactions [95]. Like plants, algae such as the marine diatom *Pseudo-nitzschia multiseries* can release L-tryptophan, which can be converted by bacteria into IAA to stimulate algal growth [96]. *Chlamydomonas* can secrete tryptophan [97], providing symbiotic microbes with the appropriate substrate for IAA production. Therefore, the role of IAA as a crosstalk molecule is not only restricted to plant–bacterial interactions, but extends to algae and is likely a widespread phenomenon in phototrophic–bacterial symbioses [96,98,99,100]. Notwithstanding, algae—including *Chlamydomonas*—can also synthesize IAA from L-tryptophan, and algal-derived auxin can also affect algal–bacterial interactions [101]. However, the role of auxin production played by the algal partner in these associations has been largely neglected. Algal-produced IAA has only recently been proposed to have a role in mutualistic interactions between *Chlamydomonas* and *Methylobacterium* spp. [102] (preprint) (Figure 1). Under N limitation, *Chlamydomonas* can produce IAA from L-tryptophan via a LAO1-mediated mechanism. The accumulation of this auxin in the medium causes the alga to arrest cell multiplication and chlorophyll degradation. This auxin-derived inhibitory effect can be relieved by the presence of *Methylobacterium* spp., which can feed on auxin to grow. Bacterial auxin degradation was promoted by the presence of the alga, which is, in turn, benefited by the reduction of auxin levels, and resumes growth. Since both *Chlamydomonas* and *Methylobacterium* spp. are found in the rhizosphere, this chemical crosstalk mediated by auxin production and degradation may impact plant fitness and could be used to improve crop yields in sustainable agriculture [102].

### 5.5. Quorum Quenching

Beyond nutrient exchange, microbial interactions are modulated by signal molecules. Using quorum sensing (QS), bacteria can sense and respond to changes in cell population density to control specific processes such as virulence factor expression, secondary metabolites production, biofilm formation, and other mechanisms involved in host–microbial interactions and microbial ecology [103]. These processes often impact their interaction with co-existing organisms and, consequently, these interacting organisms have developed the ability to interfere with bacterial quorum sensing by producing molecules that mimic QS and modulate their behavior; this process is known as quorum quenching (QQ) [104,105]. N-acyl-l-homoserine lactones (AHLs) are bacterial QS signals produced by over 50 different species [106]. The marine red alga *Delisea pulchra* secretes halogenated furanones that are structurally similar to AHL, which is the first eukaryote in which QS mimics were identified [107]. Similarly, the land plant *Medicago truncatula* can secrete more than a dozen compounds that stimulate or inhibit bacterial AHL-mediated QS [108], which suggests that QQ may be a widespread ability across photoautotrophic organisms to modulate their bacterial symbionts. In *Chlamydomonas* culture filtrates, at least a dozen AHL-mimicking molecules can stimulate QS in the soil bacterium *Ensifer meliloti* [109] (Figure 1). These AHL-like molecules have been chromatographically separated but have not been identified yet. Their production is critically dependent on the age and culture conditions; higher levels were produced under phototrophic than under mixotrophic conditions (with acetate) while the levels reduced in aged cultures. Interestingly, these molecules affect the accumulation of at least 16 *S. meliloti* proteins, including the regulatory protein PII, which is involved in the C/N balance in both prokaryotes and eukaryotes [110], and controls N assimilation and nodule formation in *S. meliloti* [111]. Since no homologs to the bacterial AHL synthases have been found in the genome of *Chlamydomonas,* the synthesis of the AHL-like molecules in *Chlamydomonas* molecules may involve different enzymes. A derivative of the vitamin riboflavin named lumichrome purified from *Chlamydomonas* culture filtrates was able to stimulate the QS LasR receptor in *Pseudomonas aeruginosa* [112] (Figure 1). This QS mimicking-signal has also been identified in rhizobial exudates that stimulate plant growth and seedling development [113]. Therefore, the production of this inter-kingdom signal molecule by *Chlamydomonas* might have a broader effect on the ecosystem, which is not only confined to bacteria, but also affects higher plants.

## 6. Harnessing *Chlamydomonas*—Microbial Interactions for Biotechnological Applications

The great metabolic diversity of microalgae, together with their rapid growth and low production costs, point to these organisms as promising resources for a wide range of biotechnological applications aimed at meeting urgent needs in industry and agriculture. For instance, microalgae have extensive potential as sustainable sources to produce bulk compounds such as biofuel, fertilizers or livestock feed, as well as for wastewater treatment and biological C sequestration [114]. Nevertheless, there are still many issues making the use of microalgae inefficient for industrial applications; thus, significant efforts are being devoted to achieving a better understanding of the biological activities related to industrial applications. The yield of many of these biotechnological processes conducted by microalgae can be increased by means of their interaction with other microorganisms, which mostly involve bacteria at present [115]. As a well-established model organism, *Chlamydomonas* has been extensively used axenically and improved with in co-culture for many of these applications that will be briefly summarized here. 

**Figure 2:**
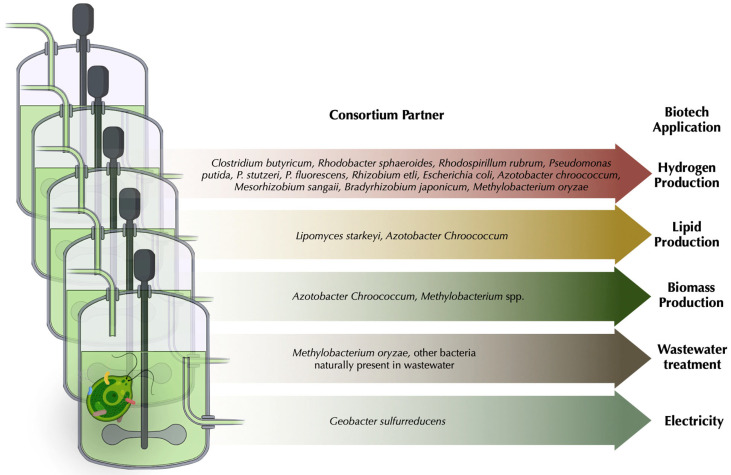
**Biotechnological applications using *Chlamydomonas*–microbial consortia**. Main applications and bacterial species used for biotechnology are depicted. For detailed information, see Section 6 in the main text. This figure was generated using Biorender.com and Keynote software.

The current climate crisis has precipitated the commitment of countries worldwide to transition from fossil fuels to alternative clean and renewable energies. Hydrogen (H_2_) is being pointed out as one of the most suitable renewable energy sources, since its combustion generates only water as product [116]. Biohydrogen production can be achieved using H_2_-producing bacteria or microalgae, while this production can be improved by their combination. The use of *Chlamydomonas* biomass can increase the efficiency of H_2_ production by different bacterial species such as *Clostridium butyricum*, *Rhodobacter sphaeroides* KD131 and *Rhodospirillum rubrum* [117,118]. In the case of *R. rubrum*, H_2_ production can be enhanced by using formate released by *Chlamydomonas* [119]. On the other hand, *Chlamydomonas* is also able to produce H_2_ by itself, which can be improved by the presence of some bacteria, as recently reviewed by Fakhimi and collaborators [120]. These bacteria include, among others, the soil bacteria *Pseudomonas putida, P. stutzeri, Rhizobium etli, E. coli* [121,122], *P. fluorescens* [123], *Azotobacter chroococcum* [124], *Mesorhizobium sangaii* [125], *Bradyrhizobium japonicum* [126], or *Methylobacterium oryzae* [127]. These bacterial-cocultures can improve *Chlamydomonas* biomass and avoid the accumulation of inhibitory waste compounds, such as oxygen or acetate, in the medium, which in turn increases H_2_ production yield [122,128]. Additionally, some bacteria can induce the accumulation of starch in *Chlamydomonas,* which may enhance H_2_ production [120,129]. Moreover, fermentative metabolites can be exchanged between bacteria and *Chlamydomonas* to improve synergic bacterial and algal H_2_ production [122,130] (Figure 2).

Microalgae have various advantages in biomass production: (i) they can be cultivated throughout the year, (ii) they do not compete for arable lands, and (iii) they are more productive per unit of land area than any plant system [131,132].

The co-culture of microalgae with other microorganisms increases growth stability and biomass productivity, and the biomass obtained can be used, for example, for biofuel production [7,133]. In *Chlamydomonas*, N deprivation greatly increases lipid accumulation, but its concomitant reduction in growth rate poses a challenge for biomass production. The diazotroph *A*. *chroococcum* can simultaneously enhance lipid and biomass productivity by promoting growth and inducing regulatory changes in the lipid metabolism of the alga [134]. The co-culture of *Chlamydomonas* with other plant/algal growth-promoting bacteria such as N-mineralizing *Methylobacterium* spp. may also be used to increase biomass, the production of algal-derived biofuels and other chemicals [135] (Figure 2). A mutualistic interaction between *Chlamydomonas* and the oleaginous yeast *Lipomyces starkeyi*, known for their oil productivity, increases lipid production and the resulting biomass was suitable for biofuel production [136]. Moreover, *Chlamydomonas* biomass can be digested by the non-reducing heterotrophic bacterium *Geobacter sulfurreducens* to convert light energy into electricity as microbial fuel cells [137] (Figure 2).

Bacteria are widely used in wastewater treatment plants, but the large O_2_ requirement is expensive. Therefore, the addition of oxygen-producing microalgae to the process has become a promising way to reduce costs in wastewater treatment. Moreover, microalgae make this process more environmentally friendly, since the bacterial release of the greenhouse gas CO_2_ is reduced by algal fixation [138,139], as recently reviewed by [140,141]. Importantly, bacteria naturally present in wastewater effluents promote the growth of microalgal species including *Chlamydomonas,* which in turn enhances the water treatment process [142]. In consortium with bacteria, *Chlamydomonas* can highly reduce the amount of N and phosphorous contaminant, and at the same time it can decrease most of the chemical oxygen demand in photobioreactors containing synthetic wastewater [127,143,144]. In addition to contaminant removal, the generated biomass can be used for lipid extraction used in biofuel production, while the remaining biomass may be applied in the generation of fertilizer or animal feed [145] (Figure 2).

Microalgae are also a great platform for the generation of high-value compounds, such as Biomimetic 3D living material [146]. The generation of a soft living matter based on cellulose has been achieved in a symbiotic relationship between *Acetobacter aceti* and *Chlamydomonas* [147]. This material was composed of bacterial cellulose produced *in situ* by *A. aceti* that benefits from the O_2_ produced by *Chlamydomonas*. The algal cells get embedded and immobilized in the cellulose gels and may be used as a new way to immobilize other commercially important microorganisms, improving product production and extraction.

## 7. Insights beyond Symbiosis

Due to the huge metabolic diversity of microalgae, a single species does not represent all microbial interactions, yet the use of *Chlamydomonas reinhardtii* provides a unique opportunity to learn about the principles that govern more complex symbioses. To date, most studies have focused on *Chlamydomonas* interactions with bacteria. Although there are some with fungi and ciliates, the potential impact on their physiology by other organisms that may co-exist with microalgae, such as viruses, plants, and animals, remains overlooked [148,149]. Although our knowledge of the metabolic exchange between microalgae and other microbes has greatly increased during the last decades, their regulation and dynamics, as well as the evolutionary drivers are only just starting to be elucidated [10,150]. Furthermore, horizontal gene transfer between microalgae and their interacting microbes may be a major driver for the evolution of new traits and their adaptation to different environments, which may improve some biotechnological applications. Moreover, the rapidly growing field of synthetic biology is critically increasing the efficiency of these processes. Current and future efforts to understand and predict microalgal–bacterial interactions will allow us to improve the control of many ecological and biotechnological processes, including biofuel production, as well as the development of new ones.

## Data Availability

All data required to evaluate the conclusions of this paper are included in the main text.
